# Development of a Low-resource TELE-assisted Home Exercise Program for Balance and Functional Mobility in Parkinson’s Disease (TELEPORT-PD): An International e-Delphi Consensus

**DOI:** 10.63144/ijt.2026.6711

**Published:** 2026-06-01

**Authors:** Arnold Fredrick D’Souza, Manikandan Natarajan, Dushyanth Babu Jasti, Auwal Abdullahi, Mohammad Al-Wardat, Mohan Ganesan, Divyani Garg, Sudhir Prabhu Haladi, Pawan Raj Pulu Ishwara, Violaine Lavoie, Francesco Lena, Selva Ganapathy Velayutham, Susan L. Whitney

**Affiliations:** 1 Department of Physiotherapy, Manipal College of Health Professions, Manipal Academy of Higher Education, Manipal, India; 2 Symbiosis College of Physiotherapy, Symbiosis International (Deemed University), Pune, India; 3 Department of Neurology, Star Hospitals, Banjara Hills, Hyderabad, India; 4 Department of Physiotherapy, Federal University Wukari, Wukari, Nigeria; 5 Department of Physiotherapy, Bayero University Kano, Kano, Nigeria; 6 Department of Rehabilitation Sciences, Faculty of Applied Medical Sciences, Jordan University of Science and Technology, Irbid, Jordan; 7 Physical Therapy Program, University of St. Augustine for Health Sciences, San Marcos, California, USA; 8 Department of Neurology, All India Institute of Medical Sciences, New Delhi, India; 9 Department of Community Medicine, Father Muller Medical College, Mangaluru, India; 10 Division of Neurology, Sheikh Shakhbout Medical City, Abu Dhabi, UAE; 11 Centre de recherche du Centre Intégré de Santé et Services Sociaux de Chaudiere-Appalaches, Québec, Canada; 12 Department of Medicine and Health, University of Molise, Campobasso, Italy; 13 IRCCS INM Neuromed, Pozzilli, Italy; 14 Department of Physiotherapy, National Institute of Mental Health and Neurosciences, Bengaluru, India; 15 Department of Physical Therapy, University of Pittsburgh, Pittsburgh, Pennsylvania, USA

**Keywords:** Balance, Delphi technique, Parkinson’s disease, Resource-limited settings, Telerehabilitation

## Abstract

Despite the recent surge in the use of telerehabilitation (TR) for neurological disorders, there is a lack of TR programs tailored to persons with Parkinson’s disease (PwPD), particularly in low-resource settings. To address this gap, we aimed to develop a tele-assisted home exercise program for improving balance and functional mobility in PwPD (TELEPORT-PD). An e-Delphi process was conducted with an international, interprofessional team of experts involved in rehabilitation of PwPD. A comprehensive pool of exercises was compiled and evaluated across three rounds of e-Delphi process. Out of 473 exercises pooled from literature and experts, 99 exercises entered the e-Delphi process after deduplication and were categorized under six domains. After consensus, the final program included 42 exercises along with dosage, progression, and safety considerations. The TELEPORT-PD protocol developed through an international, e-Delphi consensus could be adapted for its use in low-resource settings worldwide.

Neurological conditions are the leading cause of disability worldwide (Feigin et al., 2019). Among these diseases, Parkinson’s disease (PD) is now the fastest-growing neurological disorder (Dorsey, Sherer, et al., 2018). In the last two decades, the prevalence of PD has almost doubled globally to over 6 million and is projected to double again by the end of the next decade (Ou et al., 2021). Initially, PD was thought to be more prevalent in Western countries, however the trend is changing rapidly. Among all South Asian countries, India has the highest prevalence, followed by Pakistan, with a prevalence that is more than 1.5 times lower (Dorsey, Elbaz, et al., 2018). This necessitates new research specifically addressing the evolving needs of South Asia, with a focus on the unique socioeconomic characteristics of the healthcare services in the region.

As PD progresses, long-term rehabilitation is needed to optimize management and ensure functional independence among persons with PD (PwPD) (Mak et al., 2017). Center-based outpatient physiotherapy is ideal but not feasible for long-term care (Jack et al., 2010). Traditional home exercise programs are the mainstay of long-term physiotherapy management but may be limited in their effectiveness due to poor compliance, especially among older adults (Collado-Mateo et al., 2021). Poor compliance can be attributed to myriad reasons, such as lack of interest, lack of appropriate training and instruction, fear, and poor support from caregivers (Ellis et al., 2013; Rivera-Torres et al., 2019; Schootemeijer et al., 2020).

Telerehabilitation (TR) has been a developing area of physiotherapy care. It enables physiotherapists to provide exercise training remotely to participants regardless of their geographical location. It is of special interest to PwPD because mobility limitations, among other impairments, make regular hospital visits challenging. Studies have shown that TR is relatively economical compared with center-based care (Grigorovich et al., 2022; Rennie et al., 2022).

Home-based TR allows the physiotherapist to monitor and guide the performance of the home exercise program. To date, there are no TR guidelines specifically designed for the management of PD, and there is a dearth of literature from low-resource settings on home-based TR for PD.

Restoration and maintenance of balance and functional mobility are paramount to preserve functional independence and enhance quality of life in PwPD. Considering the challenges of low-resource settings like in India, a home-based TR program will be of immense help to improve compliance with long-term rehabilitation. To our knowledge, no such programs currently exist. Hence, this study aimed to develop a tele-assisted home exercise program for balance and functional mobility in PwPD living in lower-resource countries through the e-Delphi process.

## Methods

An online Delphi consensus was undertaken to develop a tele-assisted home exercise program for balance and functional mobility in PwPD (TELEPORT–PD). The study protocol was approved by the institutional ethics committee and was prospectively registered in the national clinical trials registry (CTRI/2022/09/045345).

### Expert Panel Selection

A list of potential experts was identified through ExpertScape based on research related to PwPD and TR, as well as through the professional network of the research center. Experts were considered eligible if they had a minimum of five years of clinical and/or research experience and at least one year of experience implementing TR in PwPD. Experts from various disciplines, including physiotherapy, occupational therapy, clinical psychology, speech-language pathology, community medicine, and movement disorder-specialized neurology, were selected to make the panel interprofessional. Invitations were sent to all potential experts via email, and those who agreed to participate provided electronic consent. It was emphasized to all participants that the exercises should be applicable for low-resource settings and were to be performed as part of a home-based TR program to improve balance and functional mobility in PwPD.

In addition to the exercises identified by the experts, a comprehensive list of exercises was also compiled from existing literature, including systematic reviews and clinical trials (Allen et al., 2022; Ashburn et al., 2007; Azevedo et al., 2021; Canning et al., 2015; Gobbi et al., 2009; King & Horak, 2009; Seymour et al., 2019; Truijen et al., 2022). Recommendations from existing exercise guidelines for PD and exercise protocols from published clinical trials on traditional exercise-based interventions (Ellis et al., 2017; Keus et al., 2014; Lee et al., 2025; Liguori, 2021; Osborne et al., 2022; van der Marck et al., 2014;) and TR for PD (Chen et al., 2018; Silva-Batista et al., 2024; Vellata et al., 2021) were also included. Additional information, such as dosage parameters, progression criteria, and safety recommendations, was also extracted if available. Exercises identified by the e-Delphi panel and sourced from the literature were compiled in an Excel spreadsheet, and duplicates were removed.

The list of exercises after deduplication were grouped under six domains: flexibility training, relaxation techniques, strength training, endurance training, balance training, and functional mobility training. This was done to organize the exercises for efficiency. The domains are based on pre-existing exercise categories within the literature reviewed. In round 2, the experts were provided with the list of exercises and were asked to rate each exercise on a 5-point Likert scale: “Totally Agree,” “Agree,” “Neither Agree nor Disagree,” “Disagree,” and “Totally Disagree.” Panellists were also asked to suggest modifications to the items as they deemed necessary. Exercises that received ≥70% agreement (“Totally Agree” or “Agree”) were retained for the next round, while the remaining were excluded.

The items retained for the round 3, under each of the six domains, were compiled along with dosage parameters and emailed to all participants. Experts were asked to rate each of the exercises as “Agree” or “Disagree” and provide comments on the dosage parameters.

Based on the rating, exercises with ≥70% agreement (“Agree”) were finalized. The suggestions related to dosage parameters were incorporated, and the revised draft of the exercise program was made. This revised draft of the TELEPORT-PD exercise program was sent to all the experts for the final consensus. Each round underwent pilot testing among two healthcare professionals unrelated to the study before being sent to the experts.

## Results

Email invitations were sent to 27 experts from various countries. Of these, 15 experts accepted the invitation and provided electronic consent to participate in the e-Delphi process. All the panel members selected had over five years of experience in research, academia, or clinical practice related to Parkinson’s disease, and a minimum of one year of experience in telehealth. There were no conflicts of interest between the researchers and experts who comprised the Delphi panel for this study.

The characteristics of the Delphi panel participants are summarized in Table 1.

**Figure 1:**
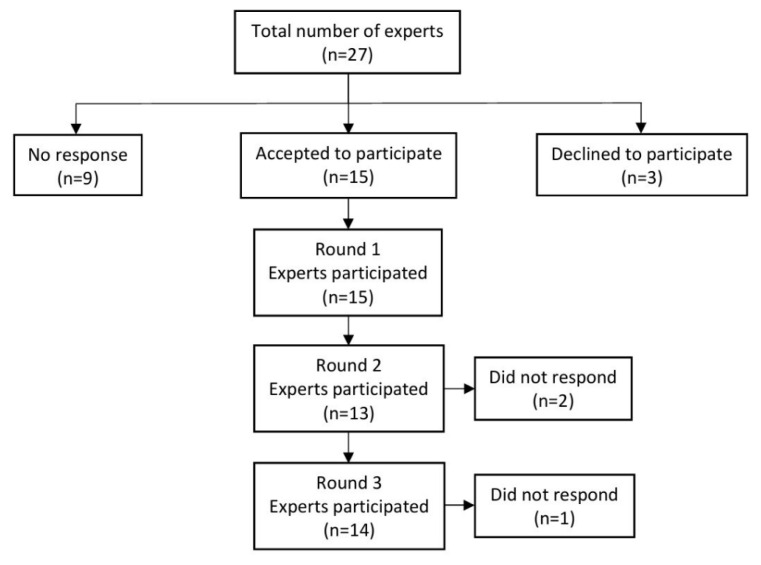
Participant Flow Diagram Showing Response Rate through the Rounds

The response rate throughout the three rounds is shown in Figure 1.

### Pooling of Exercises

The Delphi panel contributed 106 exercises, while a comprehensive literature search yielded a total of 367 exercises. After removing duplicates from the exercise list generated by both methods, 99 unique exercises were identified by the end of the first round of the e-Delphi process. Of the 99 exercises, 73 achieved the predefined level of agreement (≥70%) by the 13 panelists, who participated in round 2 of the e-Delphi process. The remaining 26 exercises were excluded, primarily due to reasons such as being less safe and limited feasibility for home-based TR, as cited by the experts.

In the final round of the e-Delphi process, responses were received from 14 panelists. Of the 73 exercises carried forward, 58 met the consensus criteria along with the dosage parameters. Further, based on panel recommendations, exercises for all the individual joints to improve the range of motion were grouped under one heading as active range of motion. Similarly, exercises to improve muscle flexibility were grouped into one category as stretching.

This reduced the total number of exercises to 42 after the final round of the e-Delphi process. This revised list of exercises was approved by the panel. The experts also agreed upon the fact that the exercise protocol can be individualized by selecting exercises that align with the participant’s specific needs and functional ability. A complete list of exercises from each round of the e-Delphi process is available from the corresponding author.

### Dosage of TELEPORT-PD

Flexibility training and relaxation techniques need to be performed daily, while exercises in the other domains need to be practiced at least 2–3 times per week on non-consecutive days. Exercise intensity will be based on Borg Rating of Perceived Exertion (RPE) scale, targeting a moderate level of exertion (i.e., 12 to 14 on 6–20 RPE scale). The duration of each exercise should be a minimum of 10 minutes, except for the flexibility and relaxation exercises, which can be as little as 5 minutes. Exercises should be discontinued, and the participant is allowed to rest if he/she experiences fatigue, pain, or any discomfort. Safety measures such as adequate support, assistive devices, a chair, or a table need to be considered for all the required exercises. To further assure safety, all exercises in the domains of functional mobility, strength and endurance training, and balance require the presence of a caregiver. All the exercises are designed in a way that each can be progressed in terms of speed, duration, repetition, resistance, hold time, or change of support surface. The final exercise list of TELEPORT-PD, categorized under six domains with corresponding progression criteria, is shown in Table 2.

Clinicians may select exercises from each domain based on individual participant needs and abilities. Exercises are progressed provided safety is ensured. For example, individuals with early-stage Parkinson’s disease (PD) may be able to perform a greater number and variety of exercises within the Balance and Functional Mobility domains, and the exercise dosage may be increased accordingly. In contrast, individuals in the middle stage of PD may require additional assistance to perform advanced tasks such as tandem standing or single-leg standing; these exercises can be deferred until sufficient confidence and stability are achieved.

Each session lasts a minimum of 30 minutes. Ideally, sessions begin with flexibility training and conclude with relaxation exercises. Balance and functional mobility exercises are performed on the same day, while strength and endurance training are paired on alternate days. A minimum of 10 minutes is allocated to each exercise domain. Sessions are conducted on alternate days to allow adequate rest and recovery. A caregiver is present throughout the sessions to provide assistance and ensure safety. Detailed program implementation, preliminary efficacy, and acceptability were reported in a two-week feasibility trial (D’Souza et al., 2025). In addition, a six-week single-blinded randomized controlled trial with a three-month follow-up has been completed and will be published elsewhere.

## Discussion

This international, interprofessional e-Delphi consensus study aimed to develop a tele-assisted home exercise program targeting balance and functional mobility in PwPD (TELEPORT-PD). The Delphi method, which brings together experts from diverse fields to synthesize evidence-based, pragmatic recommendations, is credible in developing healthcare standards for PD (Antonini et al., 2018; Ferreira et al., 2015; Jünger et al., 2017). This approach was particularly suitable given the novelty of telehealth delivery in the Indian context and the lack of region-specific, low-resource, smartphone-based TR models. As shown in the checklist included in the Appendix, the study followed the recommendations for the Conducting and REporting of DElphi Studies (CREDES) (Jünger et al., 2017).

The TELEPORT-PD program was designed to meet the long-term rehabilitation needs of community-dwelling individuals with PD who struggle with adherence to traditional, unsupervised home exercise programs.

To our knowledge, this is the first study to design a low-resource, home-based TR program for PD. The program emphasizes minimal reliance on specialized equipment, thereby enhancing accessibility in low- and middle-income countries. Accessibility is a critical factor in scaling telehealth interventions. Global standards developed by the World Health Organization and the International Telecommunication Union emphasize the need for inclusive design. This includes minimizing fine motor demands and reducing interface complexity for individuals with mobility impairments (Mantri et al., 2024).

Smartphones, which are widely available even in low-income settings, offer a practical platform for delivering TR. In India, WhatsApp Messenger is the most widely used mobile communication application, making it an ideal, low-cost medium for real-time video-based supervision and guidance (Barayev et al., 2021; Degenhard, 2025; Giordano et al., 2017).

The TELEPORT-PD program integrates exercises sourced from both in-person clinical interventions (Allen et al., 2022; Ashburn et al., 2007; Azevedo et al., 2021; Canning et al., 2015; Gobbi et al., 2009; King & Horak, 2009; Seymour et al., 2019; Truijen et al., 2022;) and home-based TR studies (Chen et al., 2018; Silva-Batista et al., 2024; Vellata et al., 2021). It also draws on established guidelines, including those from the European Physiotherapy Guideline for PD (Keus et al., 2014), the American Physical Therapy Association (Osborne et al., 2022), the American College of Sports Medicine (Ligouri, 2021), and the American Parkinson’s Disease Association (Ellis et al., 2017), as well as recommendations for fall prevention in PD (van der Marck et al., 2014). Expert contributions further enriched the exercise pool, ensuring the program reflects both evidence and clinical experience.

This multimodal program incorporates key components of fall prevention, including aerobic, resistance, balance, gait, and flexibility training (Allen et al., 2022; Hulbert et al., 2019). Exercises are simple, adaptable, and do not require advanced technology. While options like treadmill or cycling are included, alternatives such as brisk walking are provided to accommodate resource limitations.

The TELEPORT-PD program was developed through consensus among a globally diverse, interprofessional panel, with a specific focus on low-resource applicability. Although physiotherapists were overrepresented, this was intentional given their central role in exercise prescription. While feedback from PwPD was not included in the Delphi process, it was addressed in a feasibility study published elsewhere (D’Souza et al., 2025). Additionally, the findings of the single-blinded randomized controlled trial will be published, providing more empirical evidence on the program.

Finally, although designed for PD, the framework and methodology of TELEPORT-PD may inform the development of low-resource TR programs for other neurological populations with similar rehabilitation needs.

## Conclusion

The tele-assisted home exercise program for balance and functional mobility in Parkinson’s disease (TELEPORT–PD) was developed based on an international and interprofessional e-Delphi consensus process. This low-cost program is applicable to individuals in early and mid-stages of PD living in low-resource settings in India. This program enhances the accessibility of rehabilitation services in these settings.

## Figures and Tables

**Table 1:** Participant Characteristics of the Delphi Panel

Sr No	Area of work	Country	Professional expertise
1	Research, Academia, Clinical	India	Physiotherapy
2	Research, Academia, Clinical	India	Physiotherapy
3	Research, Academia, Clinical	United States	Physiotherapy
4	Research, Academia, Clinical	United States	Physiotherapy
5	Research, Clinical	Canada	Physiotherapy
6	Research, Academia, Clinical	Italy	Physiotherapy
7	Research, Academia, Clinical	Jordan	Physiotherapy
8	Research, Academia, Clinical	Nigeria	Physiotherapy
9	Research, Academia, Clinical	India	Audiology and Speech therapy
10	Research, Academia, Clinical	India	Audiology and Speech therapy
11	Clinical	United Arab Emirates	Neurology
12	Research, Academia, Clinical	India	Neurology
13	Research, Academia, Clinical	UK	Occupational therapy
14	Research, Academia, Clinical	India	Clinical Psychology
15	Research, Academia, Clinical	India	Community Medicine

**Table 2:** TELEPORT-PD Exercises by Domain with Progression Criteria

S No	Exercises	Domain	Mode of Progression
1.	Self-stretching of neck, trunk, and limb muscles	Flexibility training	Increase holding duration & repetitions
2.	Active range of movement for neck, trunk & limbs		
3.	Deep breathing	Relaxation techniques	Not applicable
4.	Progressive muscle relaxation		
5.	Front raise (shoulder flexion)	Strength training	Increase resistance and repetitions
6.	Side raise (shoulder abduction)		
7.	Biceps curl (elbow flexion)		
8.	Overhead Triceps(elbow) extension		
9.	Overhead Shoulder press		
10.	Push-ups (Wall or Counter-top)		
11.	Modified Plank (Core strengthening)		
12.	Hip abduction		
13.	Hip flexion		
14.	Hip extension		
15.	Knee flexion		
16.	Knee extension		
17.	Brisk walking	Endurance training	Increase duration and speed
18.	Jogging		
19.	Static cycling		
20.	Walking on a treadmill		
21.	Weight shifts in sitting	Balance training	Increase duration, reduce hand support, keep eyes closed, sit/stand/walk on a compliant surface
22.	Standing with feet together		
23.	Weight shifts in standing		
24.	Tandem standing		
25.	Single leg standing		
26.	Standing on a cushion		
27.	Reaching in different directions		
28.	Stepping in different directions		
29.	Walking in a line		
30.	Tandem walking		
31.	Figure of 8 walking		
32.	Obstacle walking		
33.	Bridging	Functional mobility training	Increase duration, repetitions, and speed
34.	Quadruped		
35.	Sit to Stand		
36.	Kicking in different directions		
37.	Partial squat		
38.	Spot marching		
39.	Walking with large steps		
40.	Walking with greater arm swing		
41.	Auditory and/or visual cue-assisted gait training		
42.	Dual-task gait training		

## Appendix

[Table t3-ijt-18-1-6711]

**Table A.1 t3-ijt-18-1-6711:** Recommendations for the Conducting and REporting of DElphi Studies (CREDES)

Guidance on Conducting and Reporting Delphi Studies (CREDES) Checklist	Page no.
**Rationale for Delphi Technique**	
***Justification***. The choice of the Delphi technique as a method of systematically collating expert consultation and building consensus needs to be well justified. When selecting the method to answer a particular research question, it is important to keep in mind its constructivist nature	2
**Planning and Design**	
***Planning and process***. The Delphi technique is a flexible method and can be adjusted to the respective research aims and purposes. Any modifications should be justified by a rationale and be applied systematically and rigorously	3, 4, 5
***Definition of consensus***. Unless not reasonable due to the explorative nature of the study, an *a priori* criterion for consensus should be defined. This includes a clear and transparent guide for action on (a) how to proceed with certain items or topics in the next survey round, (b) the required threshold to terminate the Delphi process and (c) procedures to be followed when consensus is (not) reached after one or more iterations	3
**Study Conduct**	
***Informational input***. All material provided to the expert panel at the outset of the project and throughout the Delphi process should be carefully reviewed and piloted in advance in order to examine the effect on experts’ judgements and to prevent bias	3
***Prevention of bias***. Researchers need to take measures to avoid directly or indirectly influencing the experts’ judgements. If one or more members of the research team have a conflict of interest, entrusting an independent researcher with the main coordination of the Delphi study is advisable	7
***Interpretation and processing of results***. Consensus does not necessarily imply the ‘correct’ answer or judgement; (non)consensus and stable disagreement provide informative insights and highlight differences in perspectives concerning the topic in question	4, 5
***External validation***. It is recommended to have the final draft of the resulting guidance on best practice reviewed and approved by an external board or authority before publication and dissemination	5
**Reporting**	
***Purpose and rationale****. *The purpose of the study should be clearly defined and demonstrate the appropriateness of the use of the Delphi technique as a method to achieve the research aim. A rationale for the choice of the Delphi technique as the most suitable method needs to be provided	6
***Expert panel***. Criteria for the selection of experts and transparent information on recruitment of the expert panel, socio-demographic details including information on expertise regarding the topic in question, (non)response and response rates over the ongoing iterations should be reported	3, 4, 5
***Description of the methods***. The methods employed need to be comprehensible; this includes information on preparatory steps (How was available evidence on the topic in question synthesised?), piloting of material and survey instruments, design of the survey instrument(s), the number and design of survey rounds, methods of data analysis, processing and synthesis of experts’ responses to inform the subsequent survey round and methodological decisions taken by the research team throughout the process	3, 4, 5
***Procedure****. *Flow chart to illustrate the stages of the Delphi process, including a preparatory phase, the actual ‘Delphi rounds’, interim steps of data processing and analysis, and concluding steps	4, 5
***Definition and attainment of consensus***. It needs to be comprehensible to the reader how consensus was achieved throughout the process, including strategies to deal with non-consensus	3, 4, 5
***Results***. Reporting of results for each round separately is highly advisable in order to make the evolving of consensus over the rounds transparent. This includes figures showing the average group response, changes between rounds, as well as any modifications of the survey instrument such as deletion, addition or modification of survey items based on previous rounds	3 ,4, 5
***Discussion of limitations***. Reporting should include a critical reflection of potential limitations and their impact of the resulting guidance	7
***Adequacy of conclusions***. The conclusions should adequately reflect the outcomes of the Delphi study with a view to the scope and applicability of the resulting practice guidance	7
***Publication and dissemination***. The resulting guidance on good practice in palliative care should be clearly identifiable from the publication, including recommendations for transfer into practice and implementation. If the publication does not allow for a detailed presentation of either the resulting practice guidance or the methodological features of the applied Delphi technique, or both, reference to a more detailed presentation elsewhere should be made (e.g. availability of the full guideline from the authors or online; publication of a separate paper reporting on methodological details and particularities of the process (e.g. persistent disagreement and controversy on certain issues)). A dissemination plan should include endorsement of the guidance by professional associations and health care authorities to facilitate implementation	6

*Note*. Source of Checklist: Jünger S, Payne SA, Brine J, Radbruch L, Brearley SG. (2017). Guidance on Conducting and REporting DElphi Studies (CREDES) in palliative care: Recommendations based on a methodological systematic review. *Palliative Medicine, 31*(8), 684–706. https://doi.org/10.1177/0269216317690685
